# Early warning score: a dynamic marker of severity and prognosis in patients with Gram-negative bacteraemia and sepsis

**DOI:** 10.1186/s12941-016-0139-z

**Published:** 2016-04-12

**Authors:** Mahableshwar Albur, Fergus Hamilton, Alasdair P. MacGowan

**Affiliations:** Department of Infectious Diseases and Medical Microbiology, Bristol Centre for Antimicrobial Research and Evaluation, Southmead Hospital, North Bristol NHS Trust-A Teaching Trust of University of Bristol, Westbury-on-Trym, Bristol, BS10 5ND UK; Department of Acute Medicine and Medical Microbiology, Southmead Hospital, North Bristol NHS Trust-A Teaching Trust of University of Bristol, Westbury-on-Trym, Bristol, BS10 5ND UK; Lead Public Health Microbiologist-South West of England, North Bristol NHS Trust, University of Bristol, Southmead Hospital, Westbury-on-Trym, Bristol, BS10 5ND UK

**Keywords:** Gram-negative bacteraemia, Early warning score, Clinical outcome

## Abstract

**Background:**

Early Warning Score (EWS) is a physiological composite score of six bedside vital parameters, routinely used in UK hospitals. We evaluated the prognostic ability of EWS in Gram-negative bacteraemia causing sepsis.

**Methods:**

We prospectively evaluated EWS as a marker of severity and prognosis in adult patients with Gram-negative bacteraemia. All adult patients with Gram-negative bacteraemia admitted to our tertiary Teaching hospital of the National Health Service in England were enrolled over 1 year period. The highest daily EWS score was recorded from 7 days before to 14 days after the date of onset of bacteraemia. The primary outcome was 28-day mortality.

**Main results:**

A total of 245 consecutive adult patients with Gram-negative bacteraemia with sepsis were enrolled. On multivariate analysis, following variables were associated with death for every single unit change (odds ratio in the brackets): higher age (1.05), lower mean arterial pressure (1.03), lower serum bicarbonate (1.08), higher EWS (1.27), higher SOFA score (1.36), hospital-onset of infection (5.43) and need for vasopressor agents (16.4). EWS on day 0, 1, 2, and average 14-day score were significantly higher in patients who died by 28 days from the onset of bacteraemia [95 % CI 0.4–0.6] *p* < 0.001. A stepwise rise in EWS and failure of improvement in EWS by 2 points 48 h after the onset of bacteraemia were associated with poor outcome.

**Conclusion:**

EWS is a simple and cost-effective bedside tool for the assessment of severity and prognosis of sepsis caused by Gram-negative bacteraemia.

**Electronic supplementary material:**

The online version of this article (doi:10.1186/s12941-016-0139-z) contains supplementary material, which is available to authorized users.

## Background

Sepsis is a leading cause of death across all age groups around the world, with an estimated incidence of 20–30 million cases each year [1]. The hospitalisation rate for sepsis has doubled in recent years in the United States from 11.6 to 24 per 10,000 population, and has even surpassed that of acute myocardial infarction [2, 3]. In the United Kingdom, the conservative annual estimates from Intensive Care National Audit and Research (ICNARC) data suggests that >100,000 cases with around 37,000 deaths occur each year as a result of sepsis, more than HIV/AIDS, prostate and breast cancer added together [4]. Blood stream infections (BSI) are an important cause of sepsis [5]. In the year 2013, 97,699 patient episodes of BSI were reported in UK and 58.3 % were caused by Gram-negative bacteria [6]. The incidence of Gram-negative BSI (GNBSI) has increased in the last 5 years in the UK compounded by increasing antimicrobial resistance [6, 7].

Sepsis is a systemic inflammatory disorder driven by infection and is the most common final pathway of death in infectious diseases [8]. Over 100 inflammation, immune and metabolic markers are known to show abnormal levels in the presence of sepsis. However none of these markers either in single or in combination can reliably grade the severity of sepsis or predict clinical outcome [9]. Therefore a reliable, cost-effective bedside test or parameter to grade the severity of sepsis to predict clinical outcome is urgently needed.

Early Warning Score (EWS) is a composite score of six bedside vital parameters: pulse rate, blood pressure (BP), oxygen saturation (SpO_2_), body temperature, respiratory rate, and mental state. EWS is recorded routinely in all inpatients admitted to national health service (NHS) hospitals in England as per National Institute of Health Care and Excellence (NICE) guidelines and it has become a standard of care in all NHS hospitals in England since 2007 [10]. There are slight modifications to grade EWS at different healthcare organisations to suit the local patient population and also to make it more user friendly.

We evaluated the utility of EWS in grading the severity of illness and predicting clinical outcome of patients with sepsis caused by GNBSI.

## Methods

### Study design, setting, and patients

We conducted a prospective, observational, non-interventional single centre cohort study in a tertiary teaching NHS hospital in England. All consecutive adult patients (>18 years) with a positive blood culture growing Gram-negative bacteria were enrolled (2011–2012). Patients were prospectively identified from the Department of Microbiology soon after the blood cultures flagging positive and the Gram stain showing the presence of Gram-negative bacteria. We excluded following patients (1) Paediatric patients (≤18 years of age), (2) any patients where cultures were deemed as contaminants, (3) any patients where follow-up to 28 days or primary outcome was not possible e.g. self-discharge or transfer to another healthcare facility.

### Data collection

All patients were evaluated, enrolled, monitored, and followed-up by the same clinical infection team including all data entry. All demographic, clinical, bacterial (identification and susceptibility data), antibiotic therapy and follow-up data were collected into a specifically designed data collection form (DCF). The results were manually entered onto the DCF on a daily basis during routine normal working hours. The data was subsequently transferred into a database created specifically for the study (Microsoft^®^ Access 2010).

### Ethics

A formal ethical approval of the study was obtained from the North Bristol NHS Trust clinical research and effectiveness department, and formal patient consent was exempted for the purposes of this study.

### Definitions

Bacteraemia was defined as polymicrobial if more than one pathogenic organism was isolated in the blood cultures. The likely source of bacteraemia was determined based on clinical assessment from the infection specialists, radiological, endoscopic, and surgical (operative theatre) findings. Time to source control was defined as an interval between time of collection of blood cultures and performing an intervention such as draining an abscess or removing an infected central venous catheter. Time to first antibiotic is defined as an interval between collection of blood cultures and receiving the first dose of antibiotic for which the isolate is tested susceptible by British Society of Antimicrobials and Chemotherapy (BSAC) in vitro method [11]. Time to appropriate therapy is defined as time to receiving the first dose of antibiotic(s) which is clinically appropriate based on susceptibility pattern, source of bacteraemia, breadth of spectrum to cover all significant isolates. Co-morbidity of patients was measured by means of age-corrected Charlson’s Co-morbidity index (CCMI) and severity of organ dysfunction was measured by using Sequential Organ Failure Assessment (SOFA) score [12, 13]. The acute physiological derangements caused by Gram-negative bacteraemia was measured by standard bedside cardio-respiratory parameters [such as blood pressure, oxygen saturation (SpO_2_) etc.] throughout inpatient stay (Additional file 1). Daily highest EWS score was recorded from 7 days prior (for hospital-onset cases only) to 14 days after the onset of GNBSI. If EWS score was not recorded as a part of the routine care in selective areas (e.g. obstetrics), we derived it based on the acute physiological parameters as enlisted earlier.

### Outcomes

Primary outcome was defined as all-cause 28-day mortality from the time of collection of blood cultures.

### Statistical analysis

All scale (quantitative) variables are reported as mean ± standard deviation (SD). The categorical variables (nominal) are reported as percentages. We used student *t* test to compare the continuous (scale) variables and *χ*^2^ test to compare the categorical variables. Mann–Whitney test was used for variables with a non-normal distribution. Differences between the groups were considered statistically significant at a *p* value of <0.05 and all tests were 2-sided with a 95 % confidence interval (CI). Multivariate analyses was carried out by using logistic regression and employed backwards stepwise variable elimination (with variable exit threshold at *p* > 0.05) to procure minimal adequate models. Survival analysis was performed by means of Kaplan–Meier curves. Receiver Operating Characteristic (ROC) analysis was performed to assess the prognostic value of EWS. All statistical analyses were performed by using SPSS 19.0 (SPSS, Chicago, IL, USA).

## Results

### Epidemiological, clinical and bacterial characteristics of the entire cohort

During the study period, 252 non-duplicate consecutive episodes of GNBSI were reported in our centre. Three of them were deemed contaminants, one patient lost to follow-up, and three were transferred to other health care centres. Therefore 245 patients were included in the final analysis of the cohort. About a third of them (35 %) were hospital-onset and two-thirds (65 %) were community-onset. The majority of GNBSI were in medical units (60 %) followed by surgical units (24 %) and the Emergency Department (11 %). *E. coli* was the most common pathogen (65.3 %) followed by *Klebsiella* spp. (12.7 %). Other bacteria identified included Enterobacteriaceae (12.7 %), non-fermenters (8.6 %) and Gram-negative anaerobes (3.7 %). 9 % of blood cultures were polymicrobial and 5 % were extended-spectrum β-lactamases (ESBL) producers. Diabetes mellitus (24 %) was the most common co-morbid medical condition followed by chronic kidney disease (23 %).

### Primary outcome

Overall the 28-day mortality in our cohort was 22.4 %, and in-patient mortality was 26.5 %.

### Source of GNBSI

The urinary tract was the most common source of bacteraemia (39 %) and the source was unknown in about 18 %. The source of GNBSI and their impact on 28-day mortality are shown in Table 1. Only 21 out of 245 (8.2 %) patients underwent source control intervention in our cohort. The nature of intervention was dependent on the source e.g. CVC line removal, draining a collection etc. Mean time to source control was 38.8 ± 46.5 h with a median of 20.9 h (range 0–179 h). Mean time to source control for patients who died before 28 days (3.82 ± 20 h) was not significantly different than those who survived (28.5 ± 51.1 h) *p* = 0.18, 95 % [CI −3.1 to 0.64].

**Table 1:** Infection source and primary outcome

	Died by day 28		Total
	No	Yes	
Infection source			
GI			
Count	25	7	32
% Within infection source	78.1	21.9	100.0
Hepatobiliary			
Count	34	4	38
% Within infection source	89.5	10.5	100.0
Lower resp. tract			
Count	12	6	18
% Within infection source	66.7	33.3	100.0
Skin and soft tissue			
Count	12	7	19
% Within infection source	63.2	36.8	100.0
Uncertain			
Count	28	15	43
% Within infection source	65.1	34.9	100.0
Urinary tract			
Count	79	16	95
% Within infection source	83.2	16.8	100.0
Total			
Count	190	55	245
% Within infection source	77.6	22.4	100.0

### Comparative findings for primary outcome

Univariate analyses of continuous and categorical variables for patients who died or survived at 28-days from the onset of GNBSI are shown in Table 2. On univariate analysis, following factors had an impact on primary clinical outcome: old age, high comorbidity score, higher SOFA score, hypotension, hypothermia, high CRP, lower time to positivity of blood cultures, time to first antibiotic, hospital-onset of BSI, requirement of oxygen and vasopressors, polymicrobial BSI, and unknown source of GNBSI.

**Table 2:** **Univariate analysis for primary outcome**

Scale variables	Alive mean ± STD	Dead mean ± STD	95 % CI	p value
Age	69 ± 17	75 ± 15	2 to 12	0.01
Charlson’s co-morbidity index	4.8 ± 2.9	6.7 ± 2.5	0.8 to 2.5	0.0001
Temperature at the time of BSI	38.3 ± 1	37.5 ± 1.4	−1.2 to −0.4	0.0001
Mean arterial pressure at onset of GNBSI	83 ± 17	73 ± 16	−16 to −6	0.0001
Oxygen saturation at onset of GNBSI	95.5 ± 3.4	93.9 ± 5.9	−2.8 to −0.3	0.015
Glasgow coma scale	14.7 ± 0.7	13.7 ± 1.8	−1.5 to −0.5	0.0001
Sequential Organ Failure (SOFA)	2.9 ± 2.3	5.8 ± 3.5	1.9 to 3.9	0.0001
EWS at the onset of GNBSI	3.4 ± 2.2	5.6 ± 2.6	1.4 to 2.8	0.0001
C-reactive protein	134 ± 104	190 ± 127	18 to 93	0.004
Serum bicarbonate	23 ± 5	20 ± 5	−3.6 to −0.6	0.007
Time interval-admission to GNBSI (days)	6.4 ± 14	10 ± 14	−0.7 to 7.8	0.1
Time interval-collection to positivity (h)	1.6 ± 0.8	1.2 ± 0.6	−0.5 to −0.1	0.001
Time to first antibiotic (h)	18.7 ± 22.8	10.7 ± 17	−13.8 to −2.3	0.006
Time to appropriate antibiotic (h)	19.6 ± 24	14.6 ± 18	−12.8 to 2.4	0.186
Time to source control (days)	1.9 ± 2.1	0.64 ± 0.8	−3.2 to 0.6	0.18

Categorical variable	Percentage	Percentage		p value
Gender: female/male	79/76	21/24		0.5
BSI onset: hospital/community	64/85	36/15		0.0001
BSI onset: out of h/routine h	81/73	19/27		0.147
Patient on oxygen/not on oxygen	57/89	43/11		0.009
On vasopressor/not on vasopressor	46/79	54/21		0.009
Polymicrobial BSI/monomicrobial BSI	55/80	45/20		0.007
ESBL positive/ESBL negative	73/78	27/22		0.7
Source of BSI: unknown/hepato-biliary	65/90	35/10		0.033

### Multivariate analysis for primary outcome

On multivariate analysis, following variables (odds ratio OR in the brackets) were associated with poor outcome: every single year increase in age (OR 1.05), every mmHg lower mean arterial pressure (OR 1.03), every unit drop in mmol of serum bicarbonate (OR 1.08), every single unit rise in EWS at the time of GNBSI (OR 1.27), every single unit rise in SOFA score (OR 1.36), hospital-onset of GNBSI (5.43) and need for vasopressor agents (OR 16.4) (Table 3).

**Table 3:** Multivariate analysis for primary outcome

Variable	Risk	95 % CI	p value
Age^a^	1.05	1.01–1.08	0.005
Mean arterial pressure^b^	1.03	1.0–1.05	0.014
Serum bicarbonate^c^	1.08	1.0–1.16	0.039
Early warning score^d^	1.27	1.04–1.56	0.02
Sequential Organ Failure Score^e^	1.36	1.12–1.65	0.002
BSI onset-hospital	5.43	2.23–12.85	0.0001
Vasopressor on day of BSI (yes)	16.4	2.07–125	0.008

^a^For each year increase in age

^b^For each mmHg fall in MAP

^c^For each mmol drop in HCO3

^d^For each unit increase in EWS score

^e^For each unit increase in SOFA score

### Systemic inflammatory response syndrome (SIRS) criteria at the time of GNBSI

**Fig. 1:**
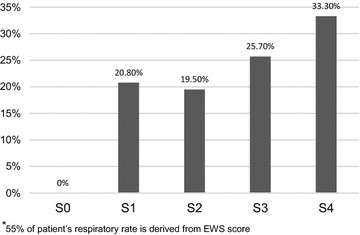
SIRS^*^ criteria of our cohort and 28-day mortality.* S0* no SIRS criteria met;* S1* 1 out of 4 SIRS criteria met;* S2* 2 out of 4 SIRS criteria met;* S3* 3 out of 4 SIRS criteria met and* S4* 4 out of 4 SIRS criteria met

In our cohort, the following proportion of patients triggered individual SIRS criteria- 63 % temperature (<36 or >38 °C); 72 % tachycardia (HR >90 bpm); 60 % abnormal white blood cell count (<4 or >12); and 28 % Respiratory rate >20 or PaCO_2_ <4.26. We did not record respiratory rate in all of our patients. However 45 % of patients had blood gas measurements giving PaCO_2_ value and in remaining 55 % of patients, we derived the respiratory rate by analysing all six components of EWS. Only 9 (3.7 %) patients had a zero SIRS score. Based on the number of SIRS criteria 0–4, there is some suggestion as to proportionate increase in mortality with each point rise in SIRS score, but this was not statistically significant (*p* = 0.25) (Fig. 1).

### Early warning score and primary outcome

EWS at the time of GNBSI, and the highest EWS on day one and day two were significantly higher in patients who died by 28 days. The mean daily highest EWS for the first 14 days from the onset of GNBSI was also significantly higher in patients who died by 28 days (Table 4). Failure of EWS to decrease by two points at 48 h was also associated with poor outcome.

**Table 4:** Early warning score changes and impact on outcome

Variable	Alive	Dead	95 % CI	p value
EWS day 0	3.4 ± 2.2	5.6 ± 2.6	1.4–2.8	<.0001
Average EWS day 0–14	1.3 ± 1.4	4.8 ± 2.7	2.8–4.3	<.0001
EWS day 1	2.0 ± 1.9	3.4 ± 2.2	0.8–2.0	<.0001
EWS day 2	1.4 ± 1.6	2.6 ± 2.1	0.7–1.9	<.0001

### Early warning score and survival analysis

**Fig. 2:**
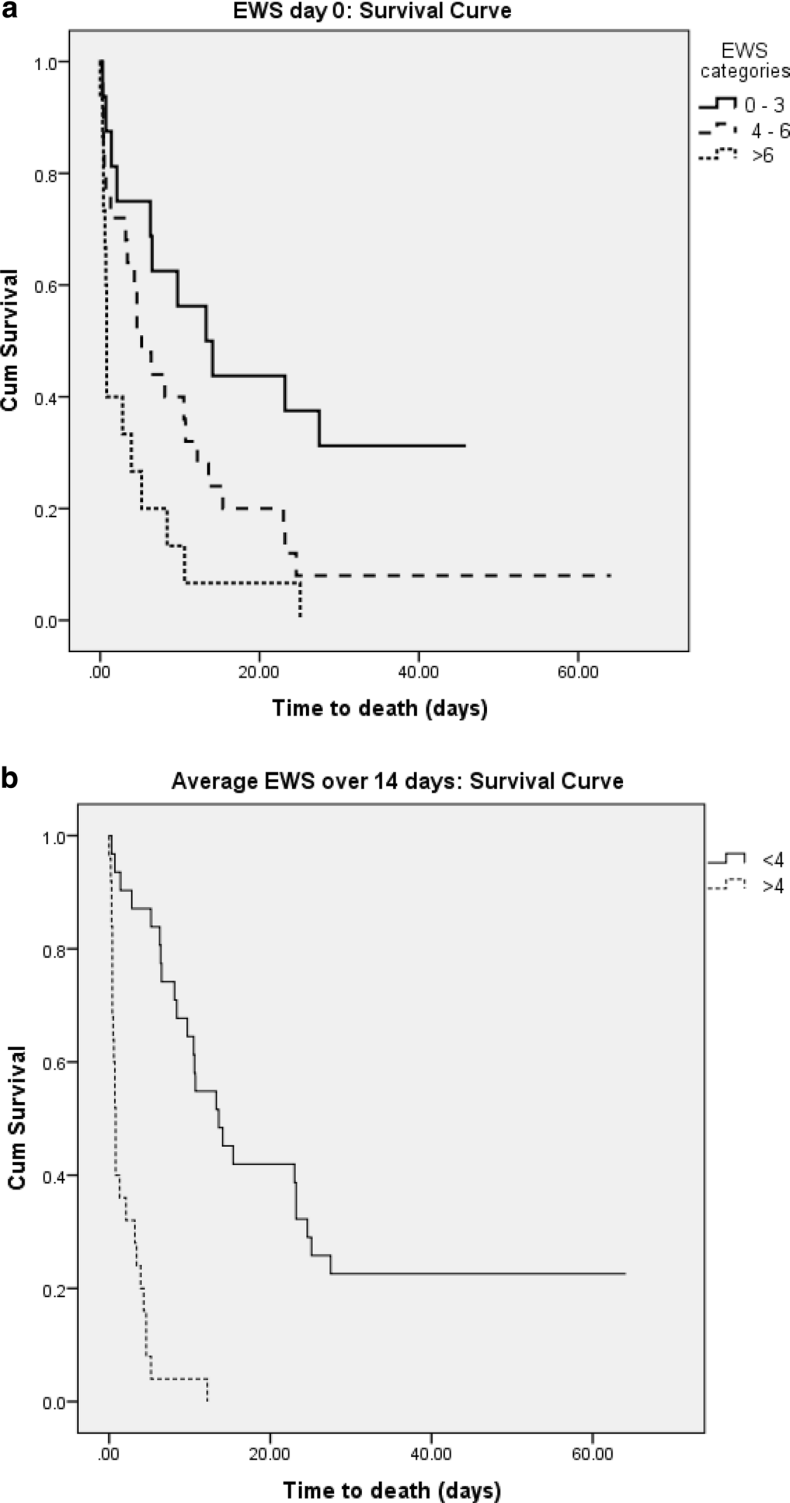
**a** Kaplan–Meier survival curve for EWS score day 0. **b** Kaplan–Meier survival curve for average EWS score over 14 days

**Fig. 3:**
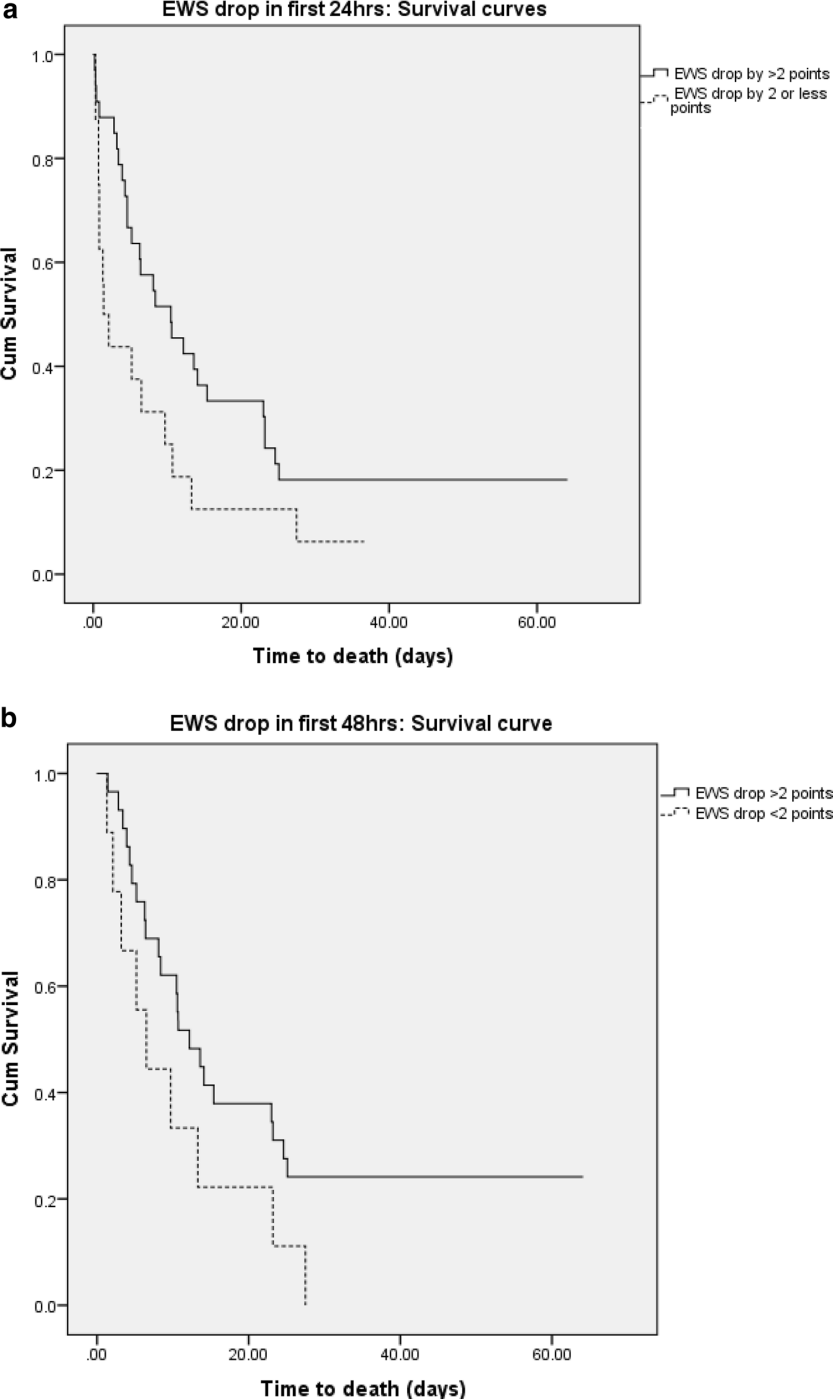
**a** Kaplan–Meier survival curve for EWS score drop within 24 h after the onset of GNBSI. **b** Kaplan–Meier survival curve for EWS score drop within 48 h after the onset of GNBSI

Kaplan–Meier survival curves indicate a clear impact of EWS on primary outcome both at the time of onset of GNBSI and average EWS for the first 14 days after onset of GNBSI (Fig. 2a, b). Higher EWS was associated with poor survival. A failure to improve EWS by two points at 24 and 48 h was also associated with poor outcome (Fig. 3a, b).

### Receiver operating characteristic (ROC) analysis of predictive value of EWS

ROC analysis was undertaken to estimate the predictive value of EWS for mortality, both at the time of bacteraemia as well as over defined time periods prior to and after bacteraemia. ROC curves were calculated for the EWS score on the day of bacteraemia, the day prior and the day after, and for the average EWS score calculated for five discrete time periods: the 7 days prior to bacteraemia, the 3 days before and after bacteraemia, the 7 days after bacteraemia, the 14 days after bacteraemia, and the whole time period (7 days before, and 14 days after). The area under the curve (AUC) for 28-days mortality is shown in Table 5.

**Table 5:** ROC analysis of predictive value of EWS

Time period	AUC (95 % CI) for 28 days mortality
EWS on day prior to bacteraemia	0.53 (0.42–0.64)
EWS on day of bacteraemia	0.72 (0.64–0.81)
EWS on day 1 after bacteraemia	0.73 (0.64–0.81)
Average EWS 7 days prior to bacteraemia	0.62 (0.53–0.70)
Average EWS 3 days before and 3 days after bacteraemia	0.79 (0.72–0.86)
Average EWS 3 days after bacteraemia	0.82 (0.75–0.89)
Average EWS 7 days after bacteraemia	0.90 (0.85–0.95)
Average EWS 14 days after bacteraemia	0.92 (0.88–0.97)
Average EWS 7 days prior, and 14 days after bacteraemia	0.89 (0.84–0.94)

## Discussion

**Fig. 4:**
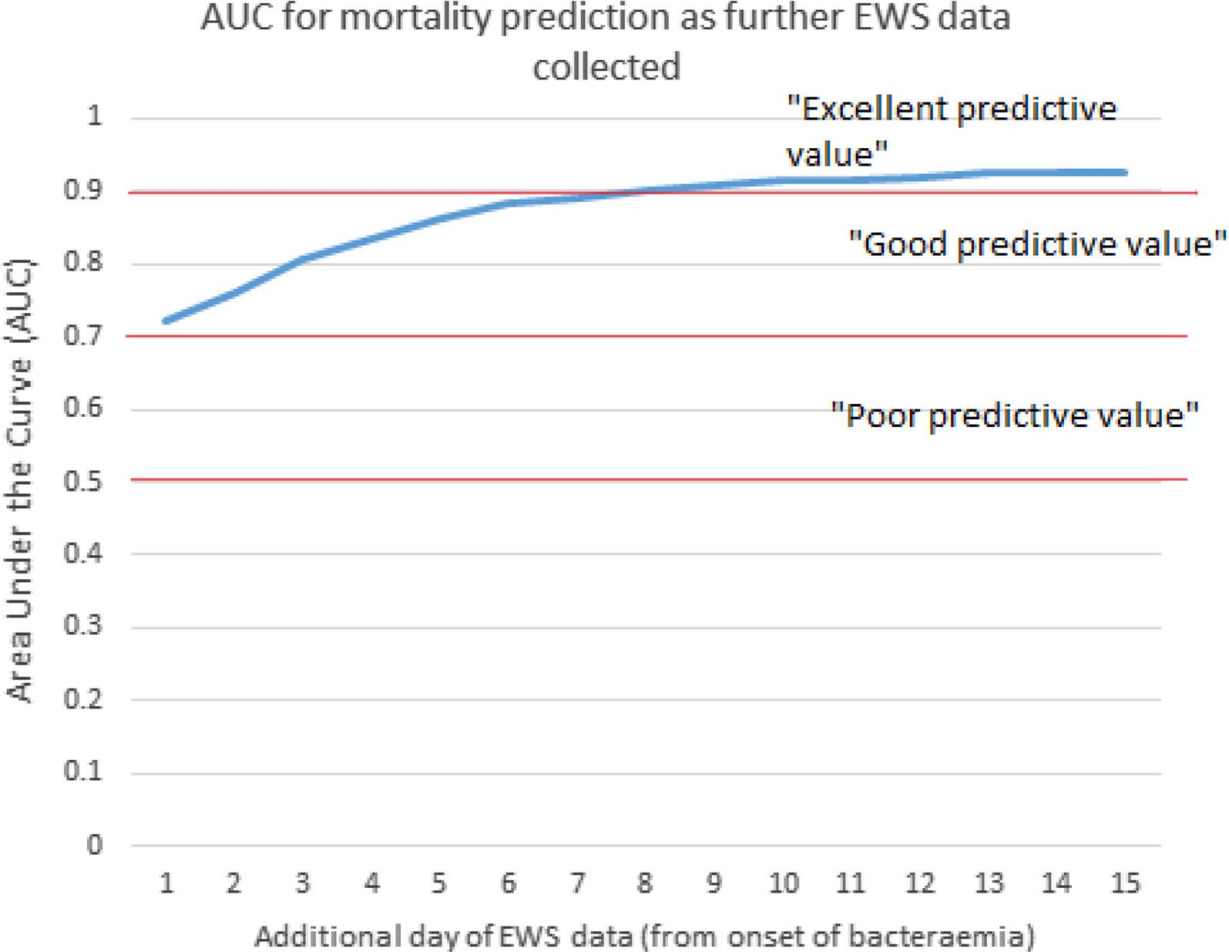
Area under the curve (AUC) for mortality prediction by EWS trend

**Fig. 5:**
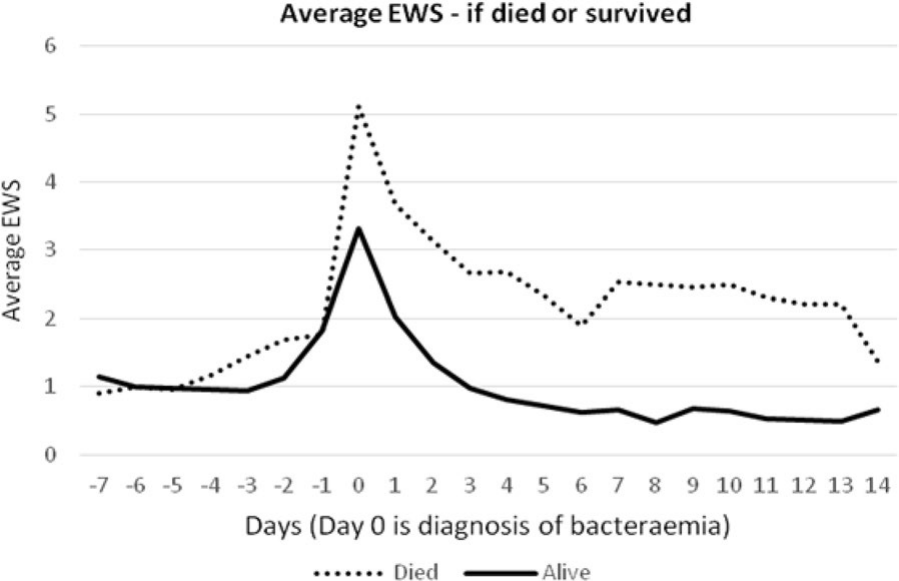
Average EWS trend and clinical outcome

Our study reiterates the multifactorial interaction and influence on clinical outcome of patients with sepsis caused by GNBSI. On multivariate analysis several factors (modifiable and non-modifiable) influenced clinical outcome including old age, low mean arterial pressure, acidosis, requirement for vasopressors, higher EWS, presence of multi-organ failure (high SOFA score), higher comorbidity score, time to appropriate antimicrobial therapy and health-care onset. Although these findings have been well described and reported in the literature, this study shows the value in terms of predicting clinical outcome by a simple bedside tool. A higher EWS at the onset and mean EWS over first 2 weeks after GNBSI with sepsis had a poor outcome. A failure to improve EWS by two points from baseline (i.e. at the onset of GNBSI) within 24 and 48 h was associated with a poor outcome. In terms of predictive value, a single EWS data at any time point on its own has a limited value, but a EWS trend over a period of time is excellent in predicting the mortality. For example, EWS score on day 0 is moderately predictive of mortality (AUC 0.72), and adding subsequent EWS data improves the models ability to predict mortality (Fig. 4). An EWS of ≥6 on day 0 was associated with a 70 % mortality, yet an average EWS of two or more throughout 14 days was associated with a 61 % mortality (Fig. 5). Also EWS showed a very limited predictive value prior to the onset of bacteraemia, but it has a very good predictive value after this event. This emphasises the fact that the derangements in acute physiological parameters as reflected in higher EWS is due to sepsis caused by GNBSI rather than comorbid conditions.

We chose to use the EWS at the time of collection of blood cultures and subsequently the daily highest value instead of an average EWS per day. This is because of the variability in the number of times the observations will be recorded between patients and the inevitable fluctuation of EWS over the course of 24 h in a given patient. Both of them can significantly skew the EWS value. Instead we wanted to focus on the most severe deranged acute physiological status per day secondary to infection, and a trend over a period of time (in days) in response to therapy. In our healthcare setting, EWS score is regularly measured for all admitted patients as per National Institute of Clinical Excellence (NICE) guidelines, at least four hourly. Any patient with an EWS score >4 due to any medical condition (not just sepsis) will trigger an emergency medical team (EMT) review patient within 30 min. Therefore patients with an EWS score (>4) at the time of collection of blood cultures in our cohort, received their antibiotic significantly earlier than patients with EWS <4 (mean difference of about 5–6 h earlier) p = 0.047 95 % CI (0.07–11.6). This is reflected in the time to initiation of appropriate antibiotic therapy amongst patients who died is paradoxically shorter than the survivors. Similarly patients with a higher EWS score (>4) had their source control procedure earlier than patients with EWS <4 (mean difference of about half-day), but this was not statistically significant *p* = 0.368; 95 % CI (−1.13 to 2.54).

A number of studies have been done in recent years assessing the relationship between EWS in sepsis (or infections) and mortality or ICU transfer [14–19]. All of these studies (except one) have assessed the prognostic ability of a single EWS value, and overall it was modest (AUC around 0.63) similar to our study (AUC 0.71) in terms of discrimination between those likely to die or survive. A single case control study from New York examining the ICU transfer of ward-based patients, showed slightly better discrimination value (AUC 0.73) using an average EWS over 72 h [20]. However, comparison between studies is difficult, as noted in a recent systematic review as most departments adapt or modify their EWS scoring criteria [21].

Serum lactate has been hailed as a surrogate marker of organ perfusion (hence severity of sepsis) and forms an important key element in the sepsis bundle [22]. However it is an invasive test with associated costs and subjected to availability especially in resource poor settings. Serum lactate is also elevated in wide variety of conditions other than sepsis [23]. A number of immune/inflammatory markers have been studied in the diagnosis, management of sepsis including assessing the severity and predicting clinical outcomes [24]. Although some of them (CRP, procalcitonin) are routinely used in clinical practice to assess the responsiveness of antimicrobial therapy, their role in assessing the severity of infection and predicting clinical outcomes is limited [25].

There are a number of sophisticated disease severity scoring systems in use especially in the critical care setting including SOFA, SAP, and APACHE II etc. In our study, SOFA score measured at the time of collection of blood cultures showed good correlation with clinical outcome. On multivariate analysis, every single point rise in SOFA score increased the mortality by 36 %. However timely measurement of these complex scoring systems at the bedside is virtually impossible even with an advanced electronic data management systems. EWS can be measured in an out of hospital settings in remote areas, and hence can be useful in resource poor countries. The major burden of sepsis and higher mortality secondary to sepsis are most prevalent in poor countries where sophisticated laboratory tests and invasive hemodynamic monitoring are unavailable [1]. Hence implementation of surviving sepsis guidelines in resource poor settings can be challenging if not impossible.

EWS is a simple, non-invasive and easy-to-repeat measurement that can be recorded by any trained healthcare worker within 1 min. EWS can be measured on multiple occasions in quick succession to follow the trend and assess the impact of interventions. Therefore EWS is an ideal marker of severity of sepsis, and dynamic changes in EWS will not only help to predict the outcome but also trigger timely interventions such as fluid balance, oxygenation, ICU transfer, and organ support. A targeted correction of EWS by means of judicious use of intravenous fluids, oxygen, and organ support such as vasopressors/inotropes may improve clinical outcome rather than focussing on blood pressure alone or relying on invasive hemodynamic monitoring such as mixed-venous saturation. We estimated the cost of measuring each EWS score as 46 pence based on time spent by the healthcare worker and the cost of the automated observation recording equipment. These costs will be even lower in a developing world because of the subsidies for the equipment and the lower salary scale. However the cost of performing a full laboratory tests including serum lactate would be significantly higher.

Our study has a few limitations, mainly in the form of number of patients enrolled and a specific type of infection (GNBSI) causing sepsis. However despite relatively low number of patients, this is a prospective study with a robust data set and having nearly a 100 % follow-up rate conducted by a dedicated infection specialist team. We deliberately chose GNBSI because significant proportions of Gram-positive bacteraemia are skin contaminants and only 3 out of 252 cases were deemed contaminants in our study. *E. coli* is the most common cause of bacteraemia in the UK, and Gram-negative bacteraemia has surpassed Gram-positive bacteraemia in the last decade or so. Also the antimicrobial resistance amongst Gram-negative bacteria is increasing and this has led to additional challenges in terms of choosing empirical therapy in patients with GNBSI until the susceptibility results are known. In our cohort, the time to first antibiotic is significantly shorter in patients who died as compared to who survived, which is in contrast to a popular retrospective study [26]. This is because, patients who died had a higher EWS score and hence were likely to have been prioritised in their care as compared to those who survived.

## Conclusion

EWS is a simple, cost-effective, and non-invasive bedside tool which is easy to record and can be repeated any number of times by a trained healthcare worker. A higher EWS at the onset and subsequent trend of sluggish decrease over 2 weeks after the onset of GNBSI is associated with poor outcome. Failure to decrease by 2 points within the first 24–48 h is also associated with poor outcome. Timely correction of EWS by judicious use of fluids and oxygenation in addition to appropriate antimicrobial therapy plus removal of focus of infection may improve clinical outcome. Large multicentre prospective randomised control studies against sepsis of any infective pathogen(s) or source is needed to explore these findings especially in resource poor settings.

## Additional file

10.1186/s12941-016-0139-z Bedside observation chart used in the study centre for recording the EWS score.

## Abbreviations

EWS: early warning score
BSI: blood stream infection
GNB: Gram negative bacteria
GNBSI: Gram negative blood stream infection
BP: blood pressure
SpO_2_: oxygen saturation
NICE: National Institute of Health Care and Excellence
NHS: National Health Service
DCF: data collection form
BSAC: British Society for Antimicrobial Chemotherapy
CCMI: Charlson’s comorbidity index
OR: odds ratio
CVC: central venous catheter
CI: confidence interval
ROC: receiver operating characteristic
ESBL: extended spectrum beta-lactamase
CRP: C-reactive protein
SD: standard deviation
SOFA: Sequential Organ Failure Assessment score
AUC: area under the curve
SIRS: systemic inflammatory response syndrome
ICU: intensive care unit
APACHE: acute physiology and chronic health evaluation

## References

[1] www.who.int, http://www.world-sepsis-day.org/CONTENTPIC/2015_WSD_FactSheet_English.pdf. Accessed 20 Oct 2015.

[2] Angus DC, Linde-Zwirble W, Lidicker J, Clermont G, Carcillo J, Pinsky MR (2001). Epidemiology of severe sepsis in the United States: analysis of incidence, outcome, and associated costs of care. Crit Care Med.

[3] Agency for Healthcare Research and Quality, U.S.G., Septicemia in US hospitals, 2009. 2011. 22049570

[4] ICNARC, Number of sepsis admissions to critical care and associated mortality, 1 April 2010 to 31 March 2013. 2013.

[5] Vincent JL, Rello J, Marshall J, Silva E, Anzueto A, Martin CD, Moreno R, Lipman J, Gomersall C, Sakr Y, Reinhart K, EPIC II Group of Investigators (2009). International study of the prevalence and outcomes of infection in intensive care units. JAMA.

[6] England PH. Health Protection Report (weekly). 2014. 8(48). https://www.gov.uk/government/statistics/escherichia-coli-e-coli-bacteraemia-annual-data. Accessed 20 Oct 2015.

[7] O’Neill J. Antimicrobial resistance review, D.o. Health, editor. London; 2014. http://amr-review.org/sites/default/files/AMR%20Review%20Paper%20-%20Tackling%20a%20crisis%20for%20the%20health%20and%20wealth%20of%20nations_1.pdf. Accessed 20 Oct 2015.

[8] Parrillo JE, Parker M, Natanson C (1990). Septic shock in humans. Advances in the understanding of pathogenesis, cardiovascular dysfunction, and therapy. Ann Intern Med.

[9] Suárez-Santamaría Marta, Santolaria F, Alemán-Valls M-R, Martínez-Riera A (2010). Prognostic value of inflammatory markers (notably cytokines and procalcitonin), nutritional assessment, and organ function in patients with sepsis. Eur Cytokine Netw.

[10] 50, N.c.g., Recognition of and response to acute illness in adults in hospital. 2007. 21204323

[11] www.bsac.org.uk, Chemotherapy, B.S.o.A. BSAC Susceptibility testing method. 2013. http://www.bsac.org.uk/wp-content/uploads/2012/02/Version-12-Apr-2013_final.pdf. Accessed 20 Oct 2015.

[12] Charlson ME, Pompei P, Ales KL (1987). A new method of classifying prognostic comorbidity in longitudinal studies: development and validation. J Chronic Dis.

[13] Vincent J, de Mendonça A, Cantraine F, Moreno R, Takal J, Suter P, Sprung C (2000). Use of the SOFA score to assess the incidence of organ dysfunction/failure in intensive care units: results of a multicenter, prospective study. Crit Care Med.

[14] Geier F, Popp S, Greve Y, Achterberg A, Glöckner E, Ziegler R, Heppner HJ, Mang H, Christ M (2013). Severity illness scoring systems for early identification and prediction of in-hospital mortality in patients with suspected sepsis presenting to the emergency department. Wien Klin Wochenschr.

[15] Ghanem-Zoubi NO, Vardi M, Laor A, Weber G, Bitterman H (2011). Assessment of disease-severity scoring systems for patients with sepsis in general internal medicine departments. Crit Care.

[16] Çıldır E, Bulut M, Akalın H, Kocabaş E, Ocakoğlu G, Aydın ŞA (2013). Evaluation of the modified MEDS, MEWS score and Charlson comorbidity index in patients with community acquired sepsis in the emergency department. Intern Emerg Med.

[17] Vorwerk C, Loryman B, Coats TJ, Stephenson JA, Gray LD, Reddy G, Florence L, Butler N (2009). Prediction of mortality in adult emergency department patients with sepsis. Emerg Med J.

[18] Barlow G, Nathwani D, Davey P (2007). The CURB65 pneumonia severity score outperforms generic sepsis and early warning scores in predicting mortality in community-acquired pneumonia. Thorax.

[19] Corfield AR, Lees F, Zealley I, Houston G, Dickie S, Ward K, McGuffie C, Scottish Trauma Audit Group Sepsis Steering Group (2014). Utility of a single early warning score in patients with sepsis in the emergency department. Emerg Med J..

[20] Yu S, Leung S, Heo M, Soto GJ, Shah RT, Gunda S, Gong MN (2014). Comparison of risk prediction scoring systems for ward patients: a retrospective nested case-control study. Crit Care.

[21] Alam N, Hobbelink E, van Tienhoven AJ, van de Ven PM, Jansma EP, Nanayakkara PW (2014). The impact of the use of the Early Warning Score (EWS) on patient outcomes: a systematic review. Resuscitation.

[22] Dellinger RP, M.M.M.L, MD2, Rhodes A, MB BS3; Annane D, MD4, et al. Surviving sepsis campaign: international guidelines for management of severe sepsis and septic shock: 2012. Crit Care Med. 2013. 41: 580–637. 10.1097/CCM.0b013e31827e83af 23353941

[23] Andersen LW, Mackenhauer J, Roberts JC, Berg KM, Cocchi MN, Donnino MW (2013). Etiology and therapeutic approach to elevated lactate. Mayo Clin Proc.

[24] Shapiro NI, Trzeciak S, Hollander JE, Birkhahn R, Otero R, Osborn TM, Moretti E, Nguyen HB, Gunnerson KJ, Milzman D, Gaieski DF, Goyal M, Cairns CB, Ngo L, Rivers EP (2009). A prospective, multicenter derivation of a biomarker panel to assess risk of organ dysfunction, shock, and death in emergency department patients with suspected sepsis. Crit Care Med.

[25] Wacker C, Prkno A, Brunkhorst FM, Schlattmann P (2013). Procalcitonin as a diagnostic marker for sepsis: a systematic review and meta-analysis. Lancet Infect Dis.

[26] Kumar A, Roberts D, Wood KE, Light B, Parrillo JE, Sharma S, Suppes R, Feinstein D, Zanotti S, Taiberg L, Gurka D, Kumar A, Cheang M (2006). Duration of hypotension before initiation of effective antimicrobial therapy is the critical determinant of survival in human septic shock. Crit Care Med.

