# Supplementary figures and images for: Early warning score: a dynamic marker of severity and prognosis in patients with Gram-negative bacteraemia and sepsis
Source: Ann Clin Microbiol Antimicrob. 2016 Apr 12;15:23. doi: 10.1186/s12941-016-0139-z (PMC4830018; doi:10.1186/s12941-016-0139-z)

**Supplementary material S1**

**
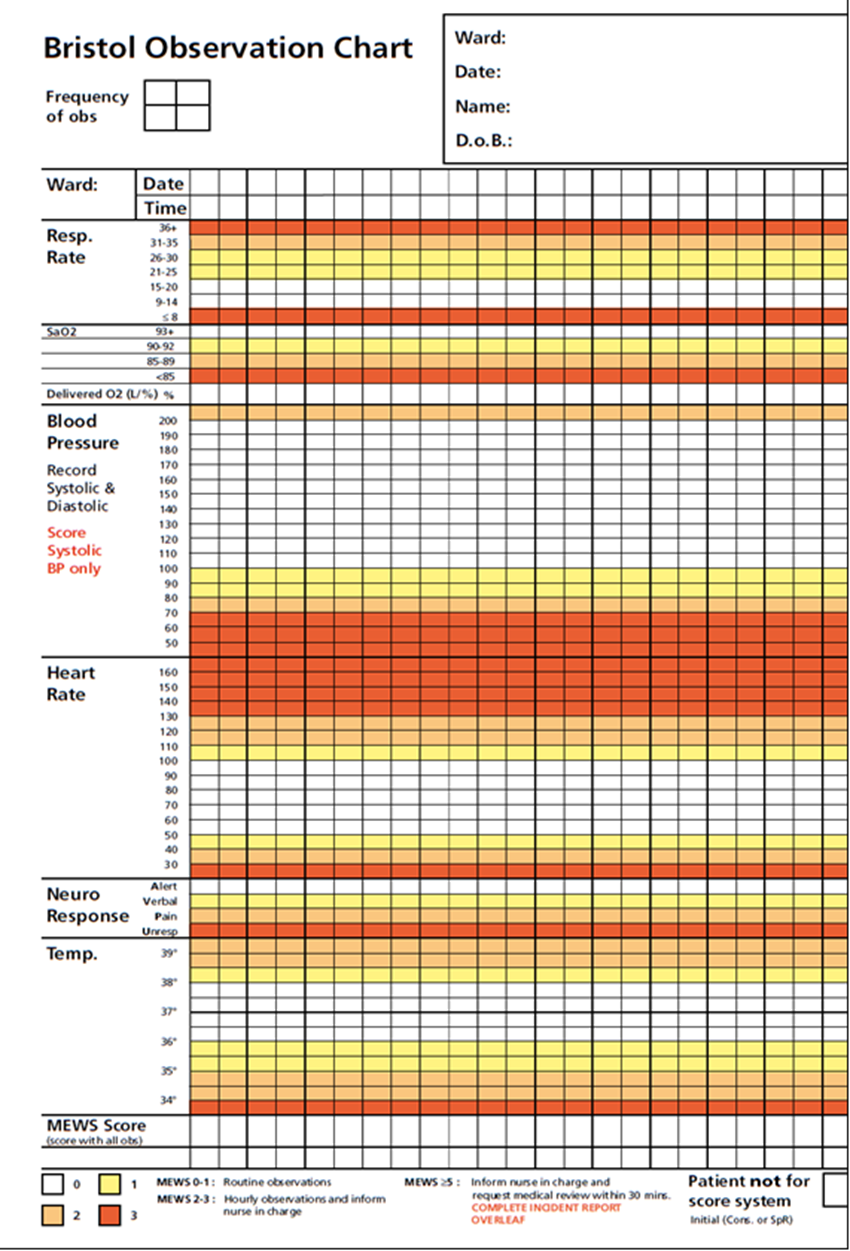
**

Supplement: Supplementary file 1 — 10.1186/s12941-016-0139-z Bedside observation chart used in the study centre for recording the EWS score. [file 12941_2016_139_MOESM1_ESM.docx]
